# ONDRI: A PROVINCIAL INITIATIVE TO UNDERSTAND THE HEALTH SYSTEM IMPACT OF AGING AND NEURODEGENERATIVE DISEASES

**DOI:** 10.1093/geroni/igz038.055

**Published:** 2019-11-08

**Authors:** Susan E Bronskill, Colleen J Maxwell, Nathalie Jette

**Affiliations:** 1 Institute for Clinical Evaluative Sciences (ICES), Toronto, Ontario, Canada; 2 University of Waterloo, Waterloo, Ontario, Canada; 3 Department of Population Health Science and Policy, Icahn School of Medicine at Mount Sinai, New York, New York, United States

## Abstract

As populations worldwide are living longer, the impact of neurodegenerative diseases on health resource utilization is expected to increase. Providing care to older adults with neurodegenerative diseases is challenging, and requires adequate supports across multiple health sectors including community, acute care and nursing home settings to allow individuals to maximize their quality of life. The Ontario Neurodegenerative Disease Research Initiative (ONDRI) is a collaborative research program that aims to improve diagnosis, treatment and management of neurodegenerative diseases including Alzheimer’s disease and related dementias, Parkinson’s disease, Amyotrophic Lateral Sclerosis (ALS), and Vascular Cognitive Impairment. Using population-based linked health administrative and clinical databases--covering over 14 million individuals residing in the province of Ontario, Canada-- the ONDRI health services research platform will address knowledge gaps regarding the health service utilization and outcomes of older adults with neurodegenerative diseases and impacts on family and care partners. Access to over two decades of historical health administrative data on a large population of older individuals uniquely positions our collaborative to examine trajectories of health system use as well as rare neurodegenerative diseases which have been previously understudied. The health services research platform is embedded within a larger ONDRI network of biomedical researchers, provincial decision-makers and health system stakeholders. Our research findings will inform health system planning and interventions to support older adults to live independently in the community. This session will explore how health administrative databases may be used to address knowledge gaps regarding health service utilization and outcomes in older persons with neurodegenerative diseases.

